# Regulating Movement in Pandemic Times

**DOI:** 10.1007/s11673-023-10292-1

**Published:** 2023-09-14

**Authors:** R. Jefferies, T. Barratt, C. Huang, A. Bashford

**Affiliations:** 1 University of New South Wales, UNSW Sydney, Sydney, NSW 2052 Australia; 2 Western Washington University, MS 9118, 516 High Street, Bellingham, Washington 98225 United States

**Keywords:** COVID-19, History, Law, Smallpox, Influenza, Border control, Pandemic

## Abstract

As COVID-19 and its variants spread across Australia at differing paces and intensity, the country’s response to the risk of infection and contagion revealed an intensification of bordering practices as a form of risk mitigation with disparate impacts on different segments of the Australian community. Australia’s international border was closed for both inbound and outbound travel, with few exceptions, while states and territories, Indigenous communities, and local government areas were subject to a patchwork of varying restrictions. By focusing on borders at various levels, our research traces how the logics of medico-legal bordering have filtered down from the international to the intra-national, and indeed, into hyper-local spaces. This is not just apparent in the COVID-19 moment but in previous pandemics of 1918 to 1919 influenza and smallpox, in which practices of quarantine and lockdowns were both unevenly distributed and implemented on multiple scales of social organization. An interdisciplinary approach between history and law reveals that human movement during pandemic times in Australia has been regulated in a manner that sees mobility as a risk to public health capable of mitigation through the strict enforcement of borders as a technology of both confinement and exclusion.

Australia’s regulation of human movement during the COVID-19 pandemic tells a story of the localization, fragmentation, and intensification of bordering practices, with unevenly distributed impacts on different segments of the Australian community. Whilst various government entities have now accepted the once-rejected notion that a full international border closure in the event of a pandemic is not only possible but effective at slowing the spread of disease (Department of Health 2020; Murphy 2020; Australian National Audit Office 2021), a close examination of sub-national entities demonstrates a similar acceptance of the effectiveness of medico-legal bordering along a continuum of localization. Our research reveals that governmental entities across all levels—from the Commonwealth, states, territories, local government areas, to Indigenous communities have approached COVID-19 (and, indeed, past pandemics) with a view of limiting movement as a primary policy response.

Our project “Rethinking medico-legal borders: From international to internal histories” began in late 2020 as a comparative analysis of instances in which laws and regulations have been implemented to restrict movement within Australia during previous pandemic episodes as well as in COVID-19. Combining legal analysis with historical research, fieldwork, and empirical investigation, this project is generating new knowledge about pandemic control and uncovering new ways of understanding two of Australia’s foundational and interconnected socio-legal problems: biosecurity and the regulation of movement. While a significant amount of legal and historical research has examined the use of international border controls as a health response, this project shifts focus away from international borders to reveal the idiosyncrasies of internal borders and the micro-level regulation of hyper-localized movement that occurs during moments of public health crisis.

Drawing on examples from COVID-19, the 1918 to 1919 influenza pandemic, and early twentieth century smallpox outbreaks, this article traces the adoption of medico-legal bordering practices at multiple scales and temporalties and offers reflections on the broader historico-legal impacts of COVID-19. We reveal how human movement during pandemic times in Australia has been regulated in a manner that sees mobility as a risk to public health that can be eliminated through the strict enforcement of borders as a technology of both confinement and exclusion, frequently occurring at highly localized levels. In our analysis of COVID-19 we highlight the agency of Indigenous communities in implementing (and advocating for) community-level border closures early in the pandemic, positioned in contrast to the onerous mandated lockdowns of “hotspot” local government areas (LGAs) which often made travel impractical or impossible for LGA residents and were applied in geographically discriminatory ways. In 1919, Indigenous communities were subject to existing governmental regulations and paternalistic control of movement, whereas local residents demonstrated varying degrees of agency over their borders, primarily invoking protective barriers rather than seeking to confine local residents. For instance, in 1903, local government officials, supported by the local health board, in Tasmania issued countless travel permits to circumvent state-imposed smallpox restrictions on movement (*Examiner* 1903a, 5). Combined, these select examples highlight the local and idiosyncratic nature of medico-legal bordering throughout Australia’s pandemic history.

Reflecting on the historico-legal impacts of COVID-19 more broadly, we note the immediate and ongoing historicization of the COVID-19 pandemic and the disruption of law and justice as a result of ongoing emergency measures. We consider the effect of disrupted temporal perceptions of the COVID-19 pandemic in the context of endemic disease and note that the events of COVID-19 have set once-unthinkable precedents for the regulation of human movement—closed borders—as the first line of defence in pandemic management. At a national scale, this meant preventing nearly all outbound travel of Australian citizens and permanent residents, whilst also barring non-Australian citizens or permanent residents from entering the country (and, in practice, many citizens as well) (Jefferies, McAdam, and Pillai 2022). At the state and territory level, this included preventing non-state or territory residents from entering without meeting strict exemptions and often requiring state or territory residents who did leave to undergo mandatory isolation or quarantine on return. At the LGA level, this included the adoption and enforcement of varying mechanisms for restricting residents’ movement both within and outside of particular LGAs.

## COVID-19

Early in the COVID-19 pandemic, remote Indigenous communities began highlighting the particular risks they faced, and some communities implemented their own border closures and requirements. For example, on 16 March 2020, the Anangu Pitjantjatjara Yankunytjatjara (“APY”) Executive Board adopted a resolution preventing travel between APY Lands and the Northern Territory, South Australia, and Western Australia, ceasing to issue new permits for travel onto APY lands, and prohibiting all non-essential travel by state and federal government employees, and employees of non-governmental organizations, amongst other restrictions (Anangu Pitjantjatjara Yankunytjatjara Executive Board 2020). The Executive Board further resolved that people travelling on APY Lands “may be stopped by the Police and asked to produce their permits and state the essential reason for their presence on the lands” (Anangu Pitjantjatjara Yankunytjatjara Executive Board 2020, 3). This closure, and similar closures by the Northern Land Council and Central Land Council (Keene 2020, 5), predated the Commonwealth government’s March 26, 2020 decision (in consultation with the Aboriginal and Torres Strait Islander Advisory Group on COVID-19) to largely restrict non-residents from entering designated remote communities in Queensland, Western Australia, South Australia, and the Northern Territory (*Biosecurity (Human Biosecurity Emergency) (Human Coronavirus with Pandemic Potential) (Emergency Requirements for Remote Communities) Determination 2020* (Cth) 2020). These successful mobilizations of Indigenous communities early in the pandemic are particularly noteworthy, given the persistent structural health inequalities and political and legal marginalization of Indigenous voices in Australia (Murray 2021).Yet, despite this early success, Indigenous communities have since been hard hit by COVID-19 due to a confluence of factors, including being disproportionately impacted by the federal government’s botched vaccine rollout (Komesaroff, et al. 2021), and the patchwork of state, territory, and federal top-down interventions (Khalil 2021; Smith 2020, 10–13).

At the LGA level, lockdowns and restrictions were generally implemented in an ad hoc approach by state or territory governments for impacted or “hotspot” LGAs. The “hotspot” term appears to have been adopted as early as April 2, 2020 by the Queensland government to describe “particular areas of Australia decided by the Chief Health Officer and published on the Queensland Health website” where there were significant numbers of COVID-19 cases (*Border restrictions Direction (No. 3) 2020* (Qld) pt 1 s 5 2020). Though generally used to describe inter-state travellers, other states, territories, and the Commonwealth later adopted this same hyper-local targeting framework, leading to divergent governance approaches. For example, National Cabinet, the intergovernmental body formed to shape the national response to COVID-19, could never agree upon a general definition of the term “hotspot” (Morrison 2020). By moving to the “hotspot model,” governments targeted specific LGAs for restrictions and closures. Though closures at hyper-local levels often did not include restrictions on residents leaving (as in the case of the Commonwealth outbound international travel restriction), distance limitations (*Public Health (COVID-19 Temporary Movement and Gathering Restrictions) Amendment (No 4) Order 2021* (NSW) 2021a; *Public Health (COVID-19 Temporary Movement and Gathering Restrictions) Amendment (No 17) Order 2021* (NSW) 2021b; *Area Directions 2020* (Vic) 2020a; *Detention Directions (33 Alfred Street, North Melbourne) 2020* (Vic) 2020b; *Stay at Home Directions (Restricted Postcodes) 2020* (Vic) 2020c; *Restrictions for Locked Down Areas (Cairns and Yarrabah) Direction 2021* (Qld) 2021; Visontay 2021), often made travel outside of a local area impractical or impossible. Furthermore, there was evidence of lockdowns being applied differently in different communities, particularly in minoritized and less wealthy parts of Sydney and Melbourne, raising questions about whether and how social factors influence hotspot risk determination (Catholic Health Australia 2022; Kaye and Gralow 2021; Victorian Ombudsman 2020).

## 1919 Influenza

The agency and authority demonstrated by residents of remote Indigenous communities during the COVID-19 pandemic, which effectively enabled communities to implement micro-level border closures, is similar to Australia’s last instance of widespread internal border closures which occurred during the 1919 influenza pandemic. In 1919, micro-level border control was not uncommon, and was routinely implemented by local councils, local “vigilance committees,” and perhaps most surprisingly, local hoteliers, all of whom had the authority to prevent outsiders from entering their space in the interest of public health (Barratt and Bashford 2022, 302–306; Moloney and Moloney 2020; Finnane 2022). For example, the residents of Meekatharra, Western Australia, effectively held up an incoming train from Perth and used the local hotel as a temporary quarantine station for the passengers, who were only allowed to enter the town after completing a week in isolation (Daily Telegraph 1919, 3). Similarly, residents of Albion Park, New South Wales, reportedly “froze out” outsiders, taking matters into their own hands by refusing travellers accommodation at their local hotels and boarding houses (Sydney Morning Herald 1919, 12). However, in 1919, Indigenous experiences of the influenza pandemic were bound up in existing systems of state and governmental regulation and control of Aboriginal movement. In Queensland for example, this resulted in flu-afflicted individuals being forcibly moved across the state, concentrating the ill in government relief depots. This approach had a devastating effect on Indigenous health, with the influenza pandemic lingering for years in these depots, while elsewhere it had all but disappeared (Briscoe 2003, 267–273). Thus, in terms of micro-level border authority, we see the reverse unfolding in 1919 and 2020, as Indigenous communities were among the few who had the power to enact their own border controls during COVID-19: LGAs, by contrast, were subject to top-down governmental controls.

## Smallpox

While some micro-level border closures were whole-heartedly embraced during the 1919 influenza pandemic, similar restrictions used to manage smallpox were challenged by the local community of Launceston, Tasmania. Smallpox, a much-feared disease that was endemic to most parts of the world but not Australia, was confirmed on June 23, 1903 in Launceston, three weeks and twelve cases after it was first noticed by local medical practitioners (Tasmanian News 1903, 4). Once alerted, the Tasmanian Central Health Board in Hobart quickly ordered the isolation of infected houses and patients, as well as the medical inspection of departing trains and their passengers. Launceston was criticized by the Central Health Board and by other states for not reporting the cases sooner, setting an antagonistic tone towards the infected city that would characterize the reception of subsequent measures (Tasmanian News 1903, 4). In addition to the delay in reporting, the local health board was slow to enact immediate directives from the state, leading Dr. John McCall from the Central Health Board to order an armed mounted patrol on all roads leading out of Launceston. Residents were forbidden from leaving unless they had an exemption pass from the mayor or a valid vaccination certificate (Examiner 1903b, 5).

Launceston locals protested the police patrol, bolstered by the vocal and physical resistance demonstrated by local officials. Alderman Saldman complained to McCall that road restrictions did not work and that even the 1900 plague outbreak in Sydney had not warranted a policed road patrol. Saldman asked “Why not have a patrol round Hobart,” given the anxiety about the disease reaching the capital (Examiner 1903b, 5). The mayor of Launceston, David Storrer, used his position to issue as many exemption passes as he could, reportedly amounting to 700–800 a day (Examiner 1903a, 5). When this did not work, Storrer led 2000 residents in a march through the medical boundary “defying the guards” (Herald 1903, 4). The next day, travellers “continued to pass out of the barrier … without forceable hindrance” from the guards (Bendigo Advertiser 1903, 4). Launceston’s local health board further appealed to have the medical border withdrawn, arguing it would do “considerable harm” and that local residents would continue to make clear their disagreement with the measures (Roe 1976, 126–135; Mercury 1903, 2).

Launceston’s reaction to state-imposed armed border controls during the smallpox pandemic contrasts with the implementation of COVID-19 restrictions. During COVID-19 in New South Wales, for example, local health boards did not drive policy decisions or impose their own restrictions on movement, though mayors and local residents called out the uneven and discriminatory impacts of the state-imposed hyper-local restrictions (Visontay and Taylor 2021; BBC News 2021; Daniel 2021). Thus, the opinions of local health boards towards micro-border regulations, whether supportive, as was mostly the case in the 1919 influenza pandemic, or oppositional, have not always been subsumed under centralized state or federal health directives as during COVID-19.

## Historical and Historico-Legal Impact

The COVID-19 pandemic has been the subject of ongoing historicization since the early months of 2020. Indeed, social commentators determined this pandemic’s status as a high-profile, profoundly impactful historic event as early as March 2020, declaring that COVID had irrevocably changed the world and writing its history in the process (Seidel 2020). Similar commentary can be found amongst legal scholars marking COVID-19’s disruption of labour law, access to justice, and other areas of law and law practice (Tham 2020; Wallace and Laster 2021). This type of social commentary, combined with frequent comparisons to other significant moments in world history, such as the 1918–1919 influenza pandemic or the Second World War, which also appeared in the news media and world leaders’ speeches since early 2020, has entangled the temporality of the COVID-19 experience (Peckham 2020). This entanglement has created a pandemic that has been consigned to history whilst it continues to unfold, where pandemic beginnings and endings are considered simultaneously, and a temporal landscape where COVID-19 past, present, and future converge.

Pandemic episodes are typically considered “over,” not when disease is eradicated but when the biological and socio-economic trajectories of disease conceptually diverge. When disease becomes endemic, social restrictions are eased and the urgency and immediacy of the pandemic threat dissipates, making it possible to return to life as normal (Charters and Heitman 2021). For example, the HIV/AIDS pandemic is considered conceptually “over,” the immediate threat historically consigned to the 1980s, while the now endemic disease continues to disproportionality ravage sub-Saharan Africa and geographically “Other” regions of the world (Greene and Vargha 2020). This illusion of endemic disease as pandemic ending creates the false perception of disease as something that does indeed end and not only disguises the true, never-ending, ongoing nature of biological disease but also prioritizes Western experiences of disease (Geissler and Prince 2020). This regrettably erases the experiences of those who continue to experience widespread suffering long after policymakers have decided that the immediate threat has passed (Kingori and McGowan 2016) and may leave in place legal options once considered unthinkable or disproportionate as a line of first response.
